# NMNAT2‐mediated NAD^+^ generation is essential for quality control of aged oocytes

**DOI:** 10.1111/acel.12955

**Published:** 2019-03-25

**Authors:** Xinghan Wu, Feifei Hu, Juan Zeng, Longsen Han, Danhong Qiu, Haichao Wang, Juan Ge, Xiaoyan Ying, Qiang Wang

**Affiliations:** ^1^ State Key Laboratory of Reproductive Medicine Nanjing Medical University Nanjing China; ^2^ Department of Obstetrics and Gynecology The Second Affiliated Hospital of Nanjing Medical University Nanjing China; ^3^ Center for Global Health, School of Public Health Nanjing Medical University Nanjing China

**Keywords:** maternal age, meiosis, metabolism, oocyte quality, oxidative stress

## Abstract

Advanced maternal age has been reported to impair oocyte quality; however, the underlying mechanisms remain to be explored. In the present study, we identified the lowered NAD^+^ content and decreased expression of NMNAT2 protein in oocytes from old mice. Specific depletion of NMNAT2 in mouse oocytes disturbs the meiotic apparatus assembly and metabolic activity. Of note, nicotinic acid supplementation during in vitro culture or forced expression of NMNAT2 in aged oocytes was capable of reducing the reactive oxygen species (ROS) production and incidence of spindle/chromosome defects. Moreover, we revealed that activation or overexpression of SIRT1 not only partly prevents the deficient phenotypes of aged oocytes but also ameliorates the meiotic anomalies and oxidative stress in NMNAT2‐depleted oocytes. To sum up, our data indicate a role for NMNAT2 in controlling redox homeostasis during oocyte maturation and uncover that NMNAT2‐ NAD^+^‐SIRT1 is an important pathway mediating the effects of maternal age on oocyte developmental competence.

**Abbreviations**
GV: germinal vesicle
GVBD: germinal vesicle breakdown
hCG: human chorionic gonadotropin
KD: knockdown
LH: luteinizing hormone
MI: metaphase I
MII: metaphase II
NA: nicotinic acid
NAD^+^: nicotinamide adenine dinucleotide
NAM: nicotinamide
Nampt: nicotinamide phosphoribosyltranferase
NMN: nicotinamide mononucleotide
Nmnat2: nicotinamide mononucleotide adenylyltransferase 2
NR: nicotinamide riboside
Pb1: first polar body
PI: propidium iodide
PMSG: pregnant mare serum gonadotropin
ROS: reactive oxygen species
Trp: tryptophan

## 1 INTRODUCTION

Mammalian oocytes undergo a protracted and discontinuous developmental programme that begins during fetal life and is not completed until postnatal adulthood (Gosden & Lee, 2010). The majority of this time is spent in a prophase I‐arrested state with an intact nucleus, termed the germinal vesicle (GV) in oocytes (Adhikari & Liu, 2014; Solc, Schultz, & Motlik, 2010). Following an extended growth phase, GV oocytes acquire the competence to resume meiosis I marked by GV breakdown (GVBD). After chromosome separation, oocytes extrude first polar body (Pb1) and are arrested at metaphase II (MII) stage awaiting for fertilization (Moor, Dai, Lee, & Fulka, 1998). Oocyte maturation is affected by a vast number of intra‐ and extra‐ovarian factors. In most mammals, oocyte quality declines with increase in maternal age (Yamamoto et al., 2010). Despite various molecules have been suggested to contribute to this process, the underlying mechanisms remain to be discovered.

Nicotinamide adenine dinucleotide (NAD^+^) is a cofactor of key enzymes in glycolysis, tricarboxylic acid cycle, and oxidative phosphorylation, participating in multiple redox reactions in cells (Camacho‐Pereira et al., 2016). Recently, the importance of NAD^+^ has expanded from a key element in intermediate metabolism to a critical regulator of multiple cell signaling pathways and now plays a major role in aging and age‐related diseases (Bonkowski & Sinclair, 2016). Sirtuins (SIRT1–7) are a family of NAD^+^‐dependent deacetylases with remarkable abilities to prevent diseases and even reverse aspects of aging (Bonkowski & Sinclair, 2016). For example, Sirt1 deficient mice showed a reduced lifespan, small size, and an increased frequency of abnormal sperm (Coussens, Maresh, Yanagimachi, Maeda, & Allsopp, 2008). In addition, sirtuins have been shown to be able to impact oocyte quality by regulating the redox state (Di Emidio et al., 2014; Kawamura et al., 2010; Liu et al., 2012; Ma, Zhang, Zhang, Han, & Rui, 2015). Loss of NAD^+^ has direct and indirect consequences on multiple cellular endpoints. In particular, depletion of intracellular NAD^+^ alters the NAD^+^/SIRT1 axis and leads to defects in mitochondrial homeostasis, reactive oxygen species (ROS) production, DNA repair, as well as cell survival (Croteau, Fang, Nilsen, & Bohr, 2017). However, to date, it remains to be determined whether NAD^+^ generation involves in oocyte aging process. In the present study, we discovered that NAD^+^ insufficiency, due to the reduced NMNAT2 expression, induces the metabolic dysfunction and meiotic defects in aged mouse oocytes.

## 2 RESULTS

### 2.1 Reduced NAD^+^ content and NMNAT2 expression in oocytes from old mice

**Figure 1:**
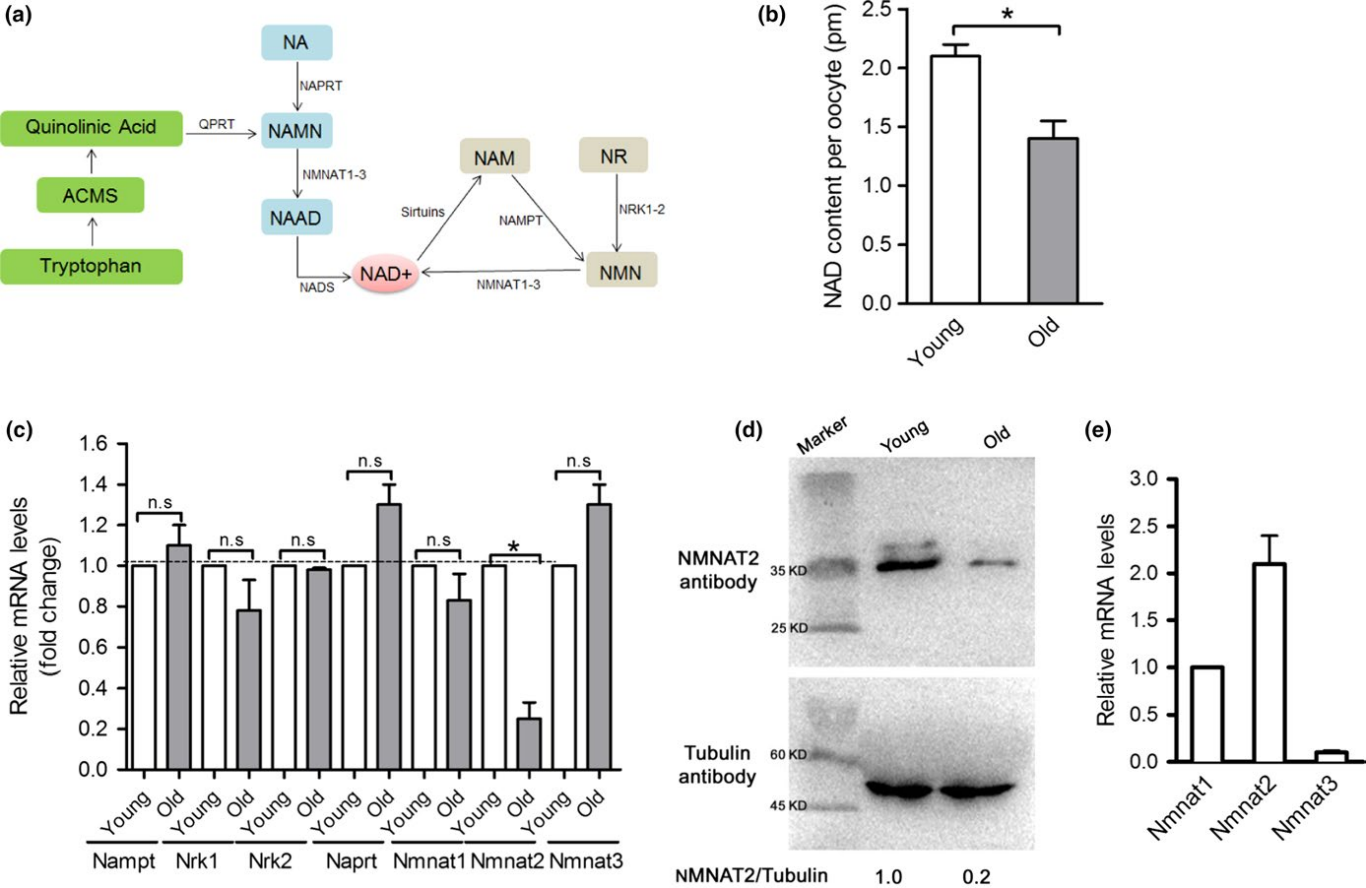
Reduced NAD^+^ content and NMNAT2 expression in oocytes from old mice. (a) NAD + is synthesized via three major pathways in mammals: (i) the de novo biosynthesis, (ii) the Preiss–Handler pathway, and (iii) the salvage pathway. (b) NAD^+^ content of oocytes from young and old mice. (c) mRNA expression of the critical enzymes responsible for NAD^+^ generation in young and old oocytes (*n* = 50 for each group). (d) Immunoblotting analysis shows the reduced NMNAT2 expression in oocytes from aged females and young controls. Band intensity was calculated using ImageJ software, the ratio of NMNAT2/Tubulin expression was normalized, and values are indicated. (e) Relative quantification of NMNAT mRNA levels in oocytes. GAPDH expression served as an internal control. Data are expressed as mean ± *SD* from three independent experiments. *p < 0.05 versus controls

In mammals, depending on the bioavailability of the precursors, there are three pathways for the synthesis of NAD^+^ in cells (Figure 1a): (a) from tryptophan (Trp) by the de novo biosynthesis pathway; (b) from nicotinic acid (NA) in the Preiss–Handler pathway; and (c) from nicotinamide riboside (NR), nicotinamide mononucleotide (NMN), and nicotinamide (NAM) in the salvage pathway (Fang et al., 2017). Mice display an age‐dependent decrease of NAD^+^ in multiple organs, including brain, muscle, pancreas, adipose tissue, and skin (Mouchiroud et al., 2013).

To determine whether the NAD^+^ production is involved in oocyte aging, we first evaluated the NAD^+^ content in oocytes isolated from young and old mice, respectively. For simplicity, herein these oocytes are termed “young oocytes” and “old oocytes,” respectively. As shown in Figure 1b, a significant decrease in the NAD^+^ level was detected in old oocytes relative to controls. Next, we checked the mRNA levels of those enzymes responsible for NAD^+^ generation by performing quantitative real‐time PCR (Figure 1c). There was no significant difference in the expression of Nampt, Naprt, Nrk1/2, and Nmnat1/3 between young and old oocytes. By contrast, the mRNA levels of Nmnat2 were dramatically decreased in old oocytes compared to controls. In line with this, we observed the lowered expression of NMNAT2 protein in oocytes from old mice (Figure 1d). It is worth noting that expression of three NMNAT members was detected (Figure 1e), with NMNAT2 mRNA being most abundant and NMNAT1 mRNA being least abundant, whereas NMNAT3 was almost undetectable, indicating that they may play differential and specific roles in oocytes. These findings suggest that the reduced NAD^+^ content and NMNAT2 expression may be associated with the impaired quality of aged oocytes.

### 2.2 Loss of NMNAT2 disturbs maturational progression and metabolic function in mouse oocytes

**Figure 2:**
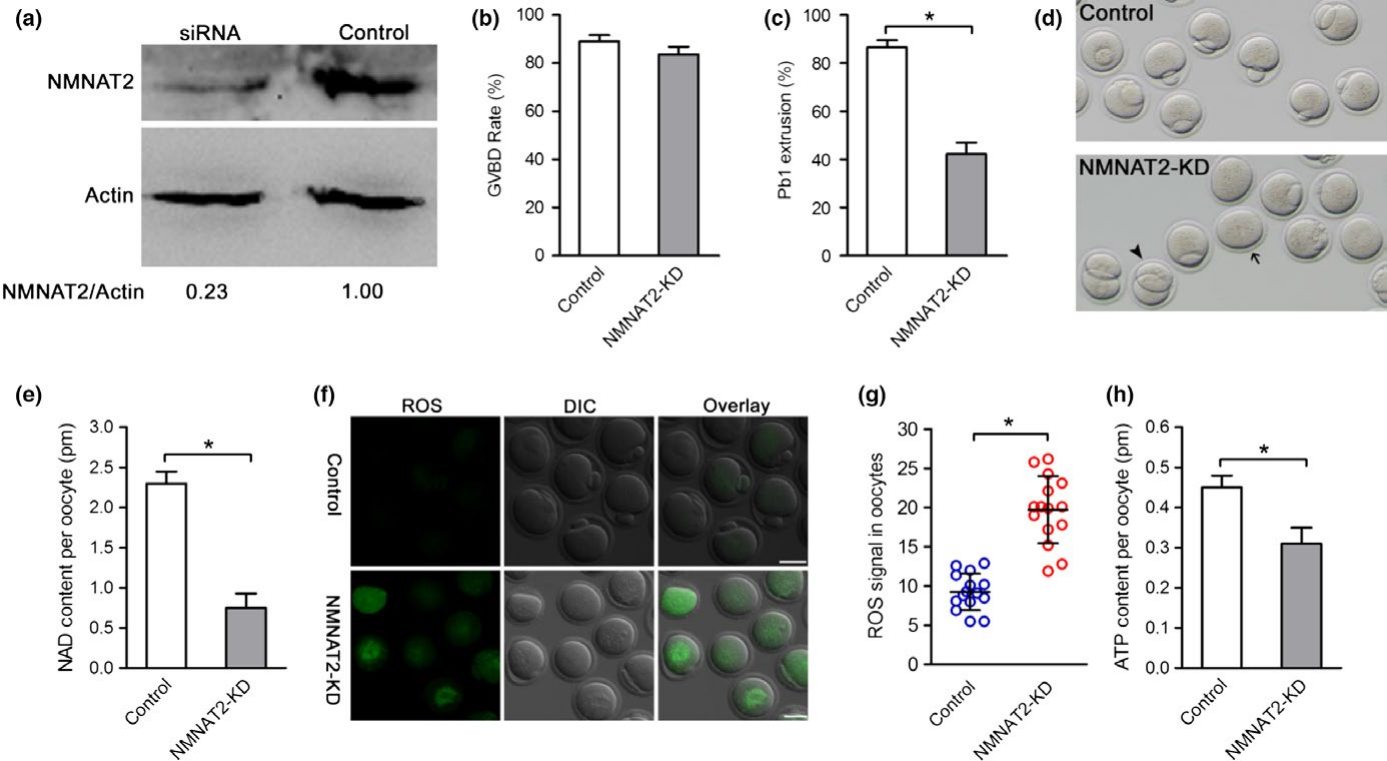
Effects of NMNAT2 knockdown on oocyte maturation. (a) The efficiency of NMNAT2 knockdown was verified by Western blot. (b,c) Quantitative analysis of GVBD and Pb1 extrusion in control (*n* = 114) and NMNAT2‐KD (*n* = 120) oocytes. (d) Phase‐contrast images of NMNAT2‐KD and control oocytes. Arrow points to oocytes that fail to extrude a polar body and arrowhead denotes the oocytes with symmetrical division. (e) Quantitative analysis of NAD^+^ content in control (*n* = 150) and NMNAT2‐KD (*n* = 150) oocytes. (f) Representative images of CM‐H2DCFDA fluorescence (green) in control and NMNAT2‐KD oocytes. (g) Quantification of ROS signals in oocytes. Values are presented as individual dot plot, *n* = 15 for each group, biologically independent oocytes from at least two different cultures. (h) Histogram showing the ATP levels in control, NMNAT2‐KD oocytes (*n* = 60 for each group). Data are expressed as mean ± *SD* from three independent experiments. *p < 0.05 versus controls. Scale bars: 50 µm

To dissect the relationship between NAD^+^/NMNAT2 depletion and oocyte phenotypes, we investigate the roles of NMNAT2 during oocyte maturation via the microinjection of siRNAs specifically targeted NMNAT2. As shown in Figure 2a, an efficient knockdown of NMNAT2 protein (NMNAT2‐KD) in oocytes was confirmed by immunoblotting. Our data showed that NMNAT2‐KD did not affect meiotic resumption, evidenced by the similar rates of GVBD after three hours in vitro culture (Figure 2b). Nonetheless, the rate of first polar body (Pb1) emission was significantly decreased in NMNAT2‐KD oocytes compared to controls (Figure 2c,d, arrow). In particular, the oocytes with symmetrical division were frequently observed when NMNAT2 was knocked down (Figure 2d, arrowhead). The results indicate that oocytes loss of NMNAT2 failed to complete maturational progression.

NMNAT family members can catalyze the synthesis of NAD^+^ both in the de novo pathway and recycling pathway. Therefore, we decided to assess whether NMNAT2 depletion also affects NAD^+^ generation in oocytes. As expected, NAD^+^ content was reduced by 60% in NMNAT2‐KD oocytes compared to their controls (Figure 2e). NAD^+^ is an essential pyridine nucleotide that serves as a cofactor and substrate for a number of critical cellular processes involved in oxidative phosphorylation and ATP production (Braidy et al., 2018). In line with this notion, we found that the reactive oxygen species (ROS) signals were markedly elevated in NMNAT2‐KD oocytes (Figure 2f,g). Accordingly, NMNAT2 depletion resulted in a ~20% reduction in bulk ATP levels compared to control oocytes (Figure 2h), indicating the impairment of metabolic function.

### 2.3 NMNAT2 knockdown induces meiotic defects during oocyte meiosis

**Figure 3:**
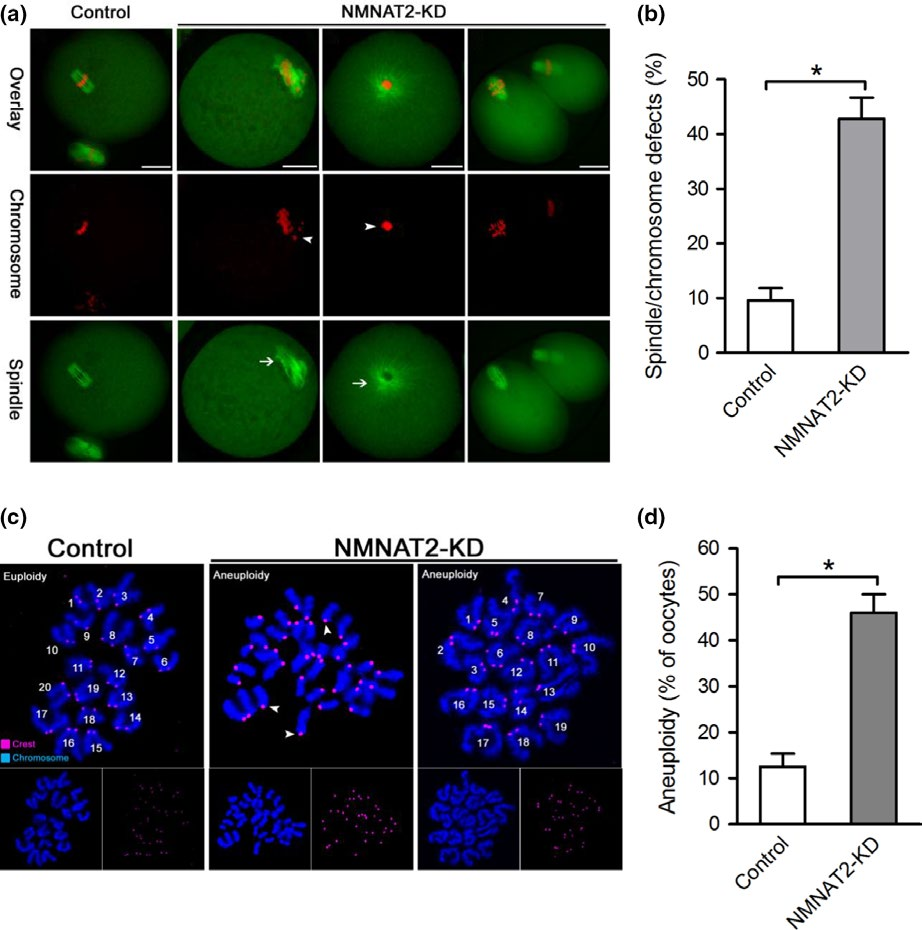
Effects of NMNAT2 knockdown on spindle assembly and chromosome alignment in oocyte meiosis. (a) Control and NMNAT2‐KD oocytes were stained with α‐tubulin antibody to visualize spindle (green) and counterstained with PI to visualize chromosomes (red). Representative confocal sections are shown. Spindle defects (arrows) and chromosomes misalignment (arrowheads) were frequently observed in NMNAT2‐KD oocytes. (b) Quantification of control and NMNAT2‐KD oocytes with abnormal spindle/chromosomes (*n* = 120 for each group). (c) Chromosome spread of control and NMNAT2‐KD MII oocytes. Chromosomes were stained with Hoechst 33342 (blue), and kinetochores were labeled with CREST (purple). Representative confocal images are shown. Arrowheads indicate the premature separation of sister chromatids. (d) Histogram showing the incidence of aneuploidy in control and NMNAT2‐KD oocytes (*n* = 20 for each group). Error bars indicate ± *SD*. *p < 0.05 versus controls. Scale bar, 25 µm

Having shown that NMNAT2 depletion disturbs meiotic progress in mouse oocytes, we asked whether NMNAT2 is required for the assembly of meiotic apparatus. To address this question, mouse oocytes from control and NMNAT2‐KD groups were stained for spindle and chromosomes organization. As shown in Figure 3a,b, we found a high frequency of spindle/chromosome defects in NMNAT2‐KD oocytes, showing the malformed spindles (arrows) with misaligned chromosomes (arrowheads). In contrast, most control metaphase oocytes displayed a typical barrel‐shape spindle and well‐aligned chromosomes on the equatorial plate.

Furthermore, to check whether these meiotic defects in NMNAT2‐KD oocytes would act to generate aneuploid eggs, we analyzed the karyotype of MII oocytes by chromosome spreading combined with kinetochore labeling. As shown in Figure 3c,d, NMNAT2 knockdown led to about 3‐fold increase in incidence of aneuploid eggs compared to control cells. In addition, the premature separation of sister chromatids in NMNAT2‐KD oocytes was also readily detected (Figure 3c, arrowheads). These results indicate that NMNAT2 knockdown induces meiotic defects in oocytes, consequently causing the generation of aneuploid eggs.

### 2.4 Nicotinic acid administration improves the oocyte quality of old mice

**Figure 4:**
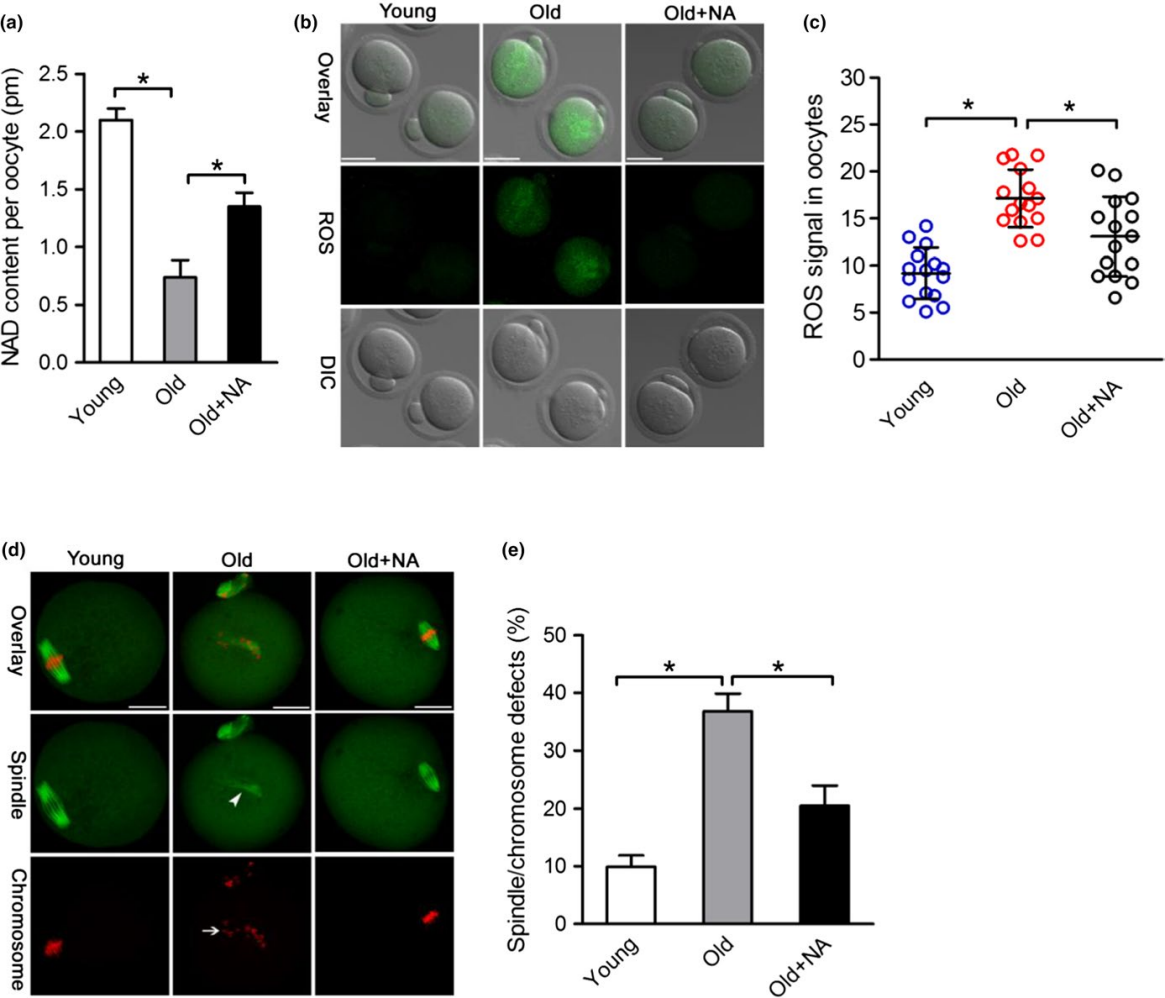
Nicotinic acid administration improves the oocyte quality of old mice. (a) Quantitative analysis of NAD^+^ content in young, old, and old + NA oocytes (*n* = 150 for each group). (b) Representative images of CM‐H2DCFDA fluorescence (green) in young, old, and old + NA oocytes. Scale bars: 50 µm. (c) Quantification of the ROS signal in oocytes. Values are presented as individual dot plot, *n* = 15 for each group, biologically independent oocytes from at least two different mice. (d) Representative images of spindle/chromosome organization in young oocytes, old oocytes, and old + NA oocytes (*n* = 120 for each group), Scale bars: 25 µm. (e) Quantification of young, old, and old + NA oocytes with abnormal spindle/chromosomes (*n* = 120 for each group). Error bars indicate ± *SD*. *p < 0.05 versus controls

Enhancing NAD^+^ biosynthesis with nicotinic acid (NA) was shown to provide significant preventive effects on various pathophysiological changes in the natural process of aging (Imai & Guarente, 2014). Hence, we investigated whether NA administration during in vitro culture could prevent the oxidative stress and meiotic defects in old oocytes. To do this, fully grown immature oocytes were retrieved from old mice and then cultured in maturation medium supplemented with or without NA. NA treatment has little effect on normal oocyte maturation (data not shown). Of note, NA supplement significantly elevated the NAD^+^ content in old oocytes, as shown in Figure 4a. Moreover, both the ROS levels and the frequency of spindle/chromosome defects were diminished in NA‐treated old oocytes (Figure 4b–e), indicating that NA administration in vitro is able to improve the quality of aged oocytes.

### 2.5 NMNAT2 overexpression ameliorates maternal age‐associated meiotic defects and oxidative stress in oocytes

**Figure 5:**
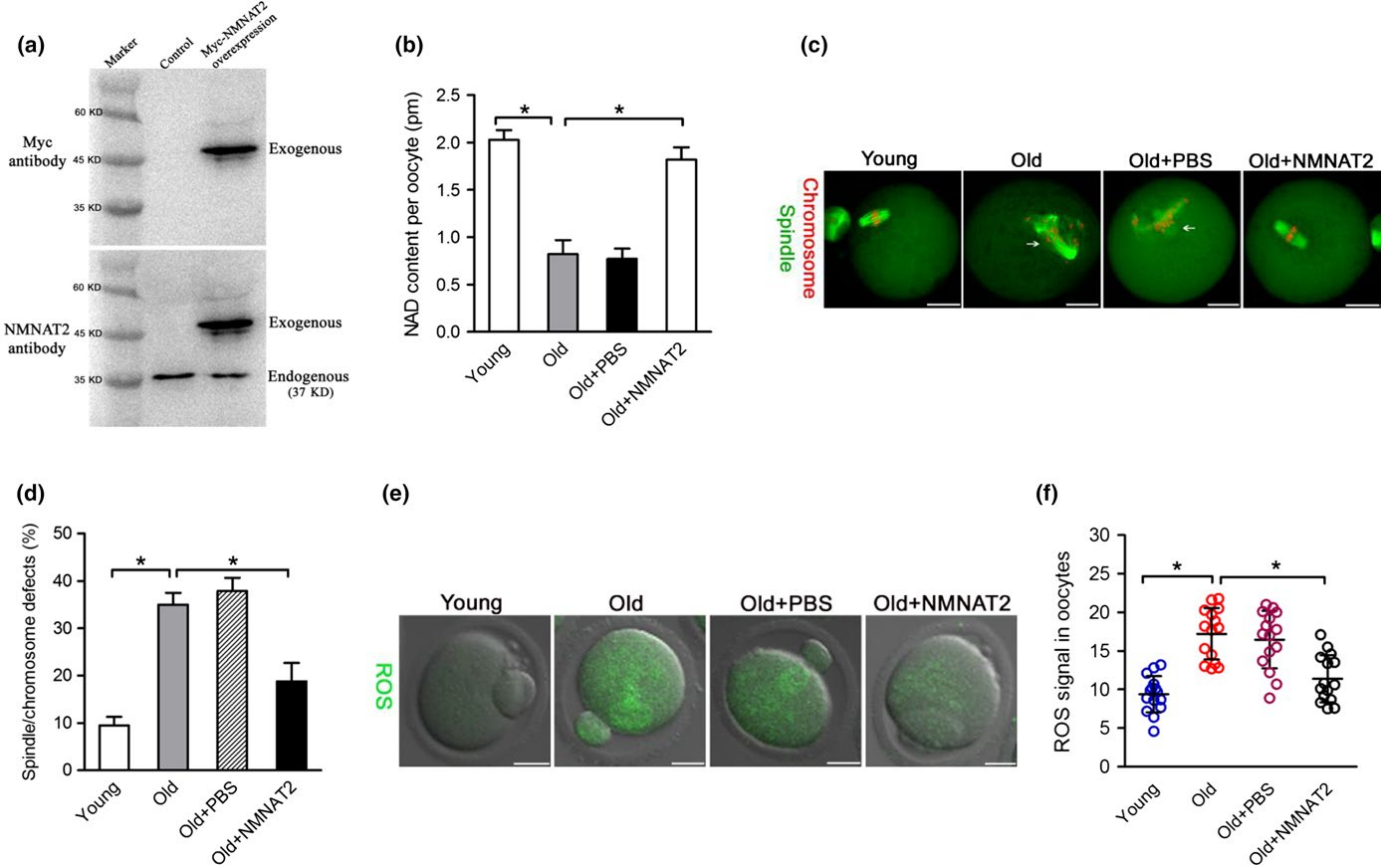
NMNAT2 overexpression ameliorates maternal age‐associated meiotic defects and oxidative stress in oocytes. (a) Western blotting analysis showing that exogenous Myc‐NMNAT2 protein was efficiently overexpressed, probing with anti‐Myc and anti‐NMNAT2 antibody. (b) Quantitative analysis of NAD^+^ levels in young, old, old + PBS, and old + NMNAT2 oocytes (*n* = 150 for each group). (c) Young, old, old + PBS, and old + NMNAT2 oocytes were stained with α‐tubulin antibody to visualize spindle (green) and counterstained with PI to visualize chromosomes (red). Representative confocal sections are shown. Spindle/chromosome abnormalities are indicated by arrows. (d) Quantification of Young, old, old + PBS, and old + NMNAT2 oocytes with spindle/chromosome defects (*n* = 120 for each group). (e) Representative images of CM‐H2DCFDA fluorescence (green) in young, old, old + PBS, and old + NMNAT2 oocytes. (f) Quantification of the ROS signals in oocytes. Values are presented as individual dot plot, *n* = 15 for each group, biologically independent oocytes from at least two different mice. Error bars indicate ± *SD*. *p < 0.05 versus controls. Scale bars: 25 µm

Given the loss of NMNAT2 in old oocytes, we checked whether elevating NMNAT2 expression in old oocytes could rescue their phenotypes. Toward this goal, exogenous Nmnat2 mRNA was injected into old GV oocytes, and then the phenotypes were assessed. Immunoblotting verified that NMNAT2 protein was efficiently overexpressed in mouse oocytes (Figure 5a). NAD^+^ levels in aged oocytes were almost restored back to normal when NMNAT was overexpressed (Figure 5b). Similarly, the phenotypic defects of old oocytes, specifically the ROS overproduction and meiotic abnormalities, were partially suppressed by the ectopic expression of NMNAT2 (Figure 5c–f). Together, these data suggest that NMNAT2 overexpression ameliorates maternal age‐associated meiotic defects and oxidative stress in oocytes.

### 2.6 SIRT1 mediates the effects of NMNAT2 on quality control of aged oocytes

**Figure 6:**
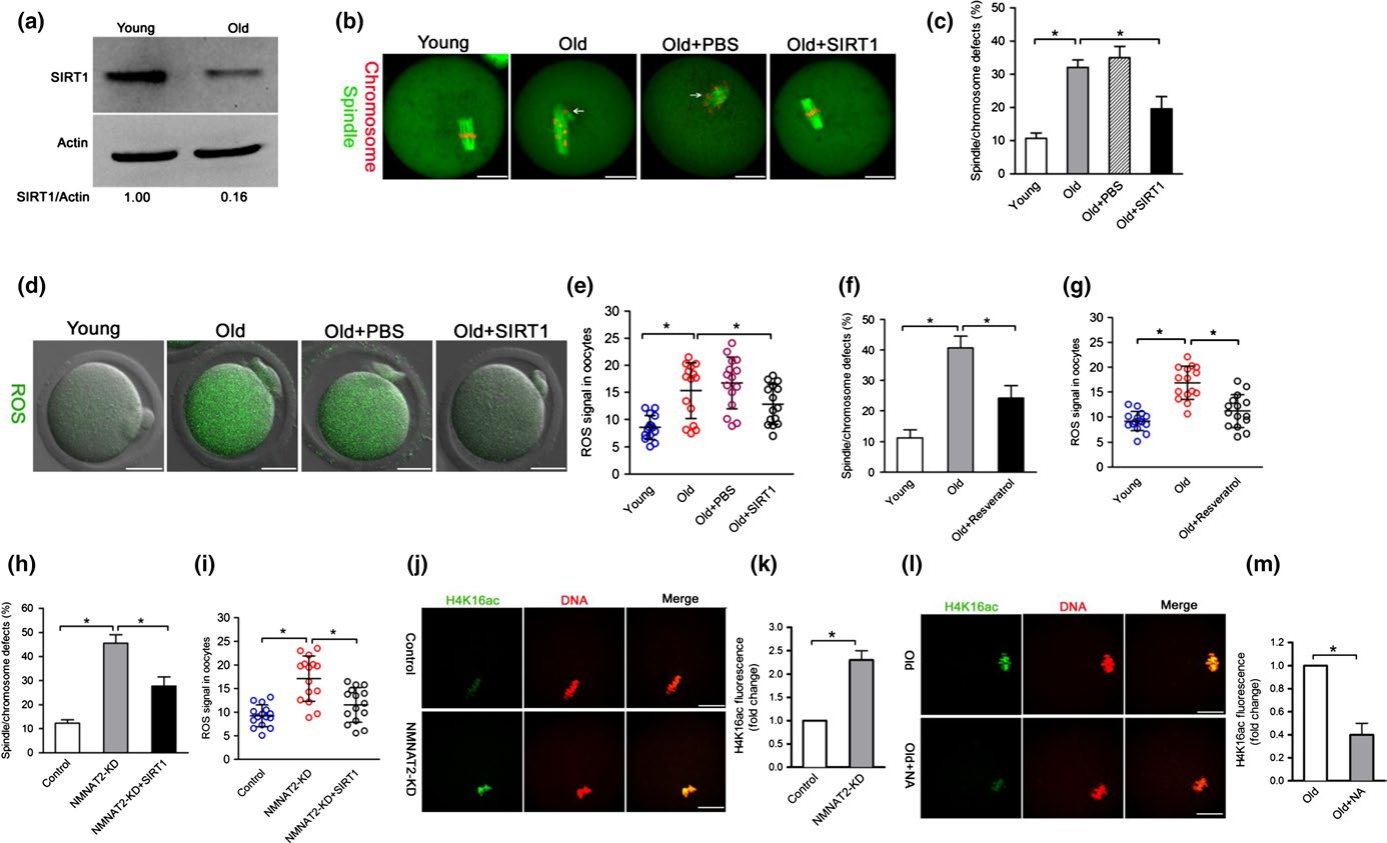
SIRT1 mediates the effects of NMNAT2 on quality control of aged oocytes. (a) Immunoblotting analysis shows the decrease SIRT1 expression in oocytes from old females compared to young controls. (b) Young, old, old + PBS, and old + SIRT1 oocytes were stained with α‐tubulin antibody to visualize spindle (green) and counterstained with PI to visualize chromosomes (red). Representative confocal sections are shown. Spindle/chromosome defects are indicated by arrows. (c) Quantification of young, old, old + PBS, and old + SIRT1 oocytes with spindle/chromosome defects (*n* = 120 for each group). (d) Representative images of CM‐H2DCFDA fluorescence (green) in young, old, old + PBS, and old + SIRT1 oocytes. (e) Quantification of the ROS signals in oocytes. Values are presented as individual dot plot, *n* = 15 for each group, biologically independent oocytes from at least two different mice. (f) Quantification of young, old, and old + resveratrol oocytes with spindle/chromosome defects (*n* = 120 for each group). (g) Quantification of the ROS signals in oocytes. Each data point represents an oocyte (*n* = 15 for each group). (h) Quantification of control, NMNAT2‐KD and NMNAT2‐KD + SIRT1 oocytes with spindle/chromosome defects (*n* = 120 for each group). (i) Quantification of the ROS signals in oocytes. Values are presented as individual dot plot, *n* = 15 for each group, biologically independent oocytes from at least two different mice. (j) Representative confocal images of acetylated‐H4K16 in control and NMNAT2‐KD oocytes. (k) Quantification of fluorescence intensity of acetylated H4K16 in control (*n* = 20) and NMNAT2‐KD (*n* = 18) oocytes. (l) Representative confocal images of acetylated‐H4K16 in old and old + NA oocytes. (m) Quantification of fluorescence intensity of acetylated H4K16 in old (*n* = 18) and old + NA (*n* = 22) oocytes. Error bars indicate ± *SD*. *p < 0.05 versus controls. Scale bars: 25 µm

Since change in NAD^+^/NADH ratio controls the activity of sirtuins, all members of this family have a crucial role in sensing the energetic status of the cell (Canto & Auwerx, 2012; Houtkooper, Canto, Wanders, & Auwerx, 2010; Yang et al., 2007). Recently, Di Emidio et al. (2014) found that SIRT1 might be involved in oocyte maturation by regulating the redox state and ensuring normal spindle assembly. It has been reported that SIRT1 activation by resveratrol enhances the biosynthesis of mitochondria in oocytes, thereby improving the developmental ability of oocytes (Sato et al., 2014). These findings prompted us to hypothesize that SIRT1 might be an important downstream mediator of NMNAT2 in controlling oocyte quality of old oocytes. To test this possibility, we first confirmed that SIRT1 protein levels were decreased by ~70% in old oocytes compared to their young controls (Figure 6a), consistent with the previous report (Di Emidio et al., 2014). Furthermore, both SIRT1 overexpression and resveratrol (a potential activator of SIRT1) treatment could alleviate the oxidative stress and meiotic defects in old oocytes (Figure 6b–g). Notably, we found that the phenotypes of NMNAT2‐KD oocytes could be partly rescued by overexpression of SIRT1 (Figure 6h,i). In addition, we also evaluated the activity of SIRT1 in oocytes by examining the acetylation status of its known target, histone H4K16 (Hajji et al., 2010; Ryall et al., 2015; Vaquero, Sternglanz, & Reinberg, 2007). As shown in Figure 6j–m, the acetylation levels of H4K16 were dramatically increased in NMNAT2‐KD oocytes compared to controls; while NA supplementation significantly reduced the acetylated H4K16 in oocytes from old mice. Altogether, these results indicate that NMNAT2 controls the quality of aged oocytes likely through modulating SIRT1 expression and/or activity.

## 3 DISCUSSION

NAD^+^ is one of the most widespread and biologically important activators within the plant and animal world. The reduced form of NAD^+^ (NADH) is a primary hydride donor in the production of ATP via anaerobic glycolysis and mitochondrial oxidative phosphorylation. NAD^+^ also serves as a co‐substrate for enzymes that create signaling metabolites or post‐translationally modify protein substrates (Frederick et al., 2016). The de novo pathway from tryptophan and the NAD^+^ biosynthetic pathway from nicotinic acid are evolutionarily conserved, while the NAD^+^ biosynthetic pathway from nicotinamide is vertebrate‐specific and mediated by NAMPT and NMNAT (Imai, 2009a) (Figure 1). In addition, NMNATs are necessary for both the Preiss–Handler pathway and the salvage pathway, playing pivotal roles in embryonic development in mice and in neuroprotection across species (Gilley & Coleman, 2010). NMNATs were initially characterized as enzymes catalyzing the reversible condensation of ATP with nicotinic acid mononucleotide (NAMN) or nicotinamide mononucleotide (NMN) to produce nicotinic acid adenine dinucleotide (NAAD) or NAD^+^ (Ali, Li‐Kroeger, Bellen, Zhai, & Lu, 2013). It has been shown that NMNAT‐2 knockdown significantly reduces cellular NAD^+^ levels and protects cells from p53‐dependent cell death upon DNA damage (Pan et al., 2014).

In the present study, we found that NAD^+^ content was decreased in oocytes from aged mice. Interestingly, we further noticed that NMNAT2 is the only NAD^+^ biosynthase enzyme showing the reduced expression in old oocytes (Figure 1). These findings indicate that NMNAT2 might be the critical enzyme responsible for NAD^+^ generation during mouse oocyte maturation. In support of this, NAD^+^ level in NMNAT‐KD oocytes was dramatically decreased as compared to control cells (Figure 2). Furthermore, NMNAT2 depletion in oocyte resulted in spindle defects and chromosome misalignment, consequently inducing the high frequency of aneuploidy (Figures 2 and 3). Any error in spindle/chromosome organization can lead to the failure of meiosis so that the oocyte cannot mature, which in humans is a major cause of pregnancy loss and developmental disabilities (Hassold & Hunt, 2001). NMNAT2‐KD oocytes showed the very similar phenotypes as aged oocytes. Female fertility decreases with advanced maternal age largely due to the meiotic abnormality in oocyte. The NAD^+^ biosynthetic pathway from NA is evolutionarily conserved. It has been shown that small amounts of NA can be converted to NAD^+^ in the intestine and liver (Fang et al., 2017). Here, we found that NA administration in vitro not only elevated the NAD^+^ content but also partly rescued the pathological phenotype of aging oocytes (Figure 4). Interestingly, it appears that NR/NMN administration has no dramatic effects on NAD level in oocytes from aged mice (data not shown). This observation indicates that old oocytes may have the compromised ability to utilize the precursors other than NA. The underlying mechanism remains to be determined. Of note, overexpression of NMNAT2 was able to alleviate the meiotic defects and oxidative stress in old oocytes (Figure 5). Altogether, based on these findings, we conclude that NMNAT2‐mediated NAD^+^ generation is essential for quality control of aged oocytes. In addition, due to the scarce amount of material and technical reason, NAD^+^ content was evaluated based on a colorimetric assay in the present study. Using a more sensitive analytical method (e.g., LC‐MS) would be helpful for the accurate quantification of oocyte NAD^+^ in the future.

**Figure 7:**
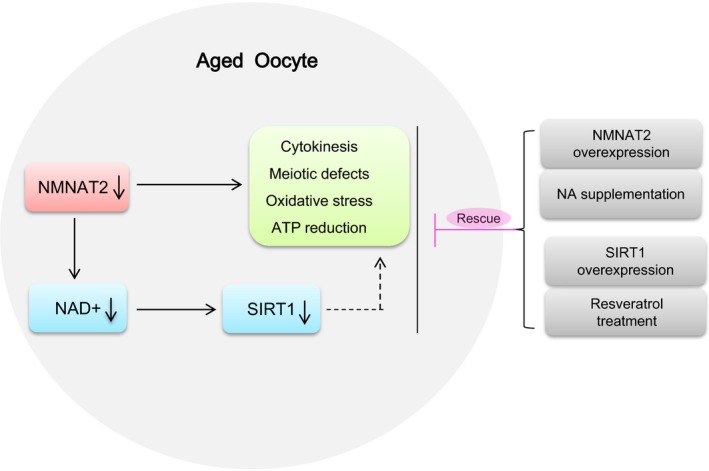
Diagram illustrating the proposed pathway mediating the effects of NAD^+^ generation on the quality control of aged oocytes. Loss of NAD^+^ content and NMNAT2 protein results in the meiotic abnormalities and metabolic dysfunction in oocytes from old mouse. NA supplement and SIRT1 overexpression/activation could partly rescue the defective phenotype of these aged oocytes

The functional connection between NAD^+^ and sirtuins is regulated at three levels: (a) regulation of NAD^+^ biosynthesis, (b) modulation of sirtuin activity/expression by NAD^+^ substrates and derivatives, and (c) competitive utilization of NAD^+^ between sirtuins and other NAD^+^ consumers (Imai & Guarente, 2016). Of them, SIRT1 serves as a universal mediator that executes metabolic effects in a tissue‐dependent manner in response to changes in systemic NAD^+^ pool (Imai, 2009b). In the present study, we showed that both elevating SIRT1 expression and SIRT1 activator could reduce the penetrance of maternal age‐associated defects in oocytes. Moreover, overexpression of SIRT1 is also capable of partially preventing those phenotypes of NMNAT‐KD oocytes. In particularly, NMNAT2 depletion and NA supplementation significantly alter the activity of SIRT1 in oocytes (Figure 6). Collectively, it is tempting to propose that NAD^+^/SIRT1 axis is an important pathway mediating the effects of NMNAT2 on oocyte quality control (Figure 7).

A hallmark of animal development is an age‐related decrease in fertility. This is largely attributed to females producing eggs of compromised developmental competence. Our study indicates a novel mechanism controlling oocyte development of aged mice, which opens a new area for assessing oocyte quality as well as clinical management of fertility issue.

## 4 MATERIALS AND METHODS

All chemicals and culture media were purchased from Sigma (St. Louis, MO, USA) unless stated otherwise.

### 4.1 Mice

ICR female mice (3–4 weeks old) were used in our experiments. To generate a natural aging model, 42‐ to 45‐week‐old female mice (near the end of their reproductive lifespan) were selected. Mice were maintained in alternating 12‐hr light/dark cycles. All animal experimental protocols were performed in accordance with relevant ethical guidelines and regulations, and approved by the Animal Care and Use Committee of Nanjing Medical University.

### 4.2 Antibodies

Mouse polyclonal anti‐SIRT1 antibodies (Cat#: S5196), anti‐H4K16ac antibodies (Cat#: ab109463), and mouse monoclonal anti‐NMNAT2 antibodies (Cat#: ab56980) were purchased from Abcam (Cambridge, MA, UK); Myc antibodies were purchased from Cell signaling Technology (Cat#: 2278); mouse monoclonal FITC‐conjugated anti‐α‐tubulin antibodies (Cat#: F2168) and mouse monoclonal anti‐β‐actin antibodies (Cat#: A5441) were purchased from Sigma; Cy5‐conjugated goat anti‐human IgG was purchased from Jackson Immuno Research Laboratory (West Grove, PA, USA).

### 4.3 Oocyte collection and culture

Oocytes were isolated from female mice at the age of 3–4 weeks (young mice) and 42–45 weeks (reproductively old mice). To collect fully grown GV oocytes, mice were superovulated with 5 IU Pregnant Mares Serum Gonadotropin (PMSG). After 48 hr, cumulus–oocyte complexes were obtained by manually rupturing of antral ovarian follicles. Cumulus cells were removed by repeatedly mouth‐pipetting. For in vitro maturation, GV oocytes were cultured in M16 medium at 37°C in an atmosphere of 5% CO_2_. For in vitro supplement, fully grown oocytes from old mice were cultured in maturation medium containing 50 μM of nicotinic acid (Sigma, St. Louis, MO) (Huang et al., 2014; Stach et al., 2012) or 2 μM of resveratrol (Ma et al., 2015).

### 4.4 Plasmid construction and mRNA synthesis

Total RNA was extracted from 100 mouse oocytes using Arcturus PicoPure RNA Isolation Kit (Applied Biosystems, CA, USA), and the cDNA was generated with QIAquick PCR Purification Kit (Qiagen, Germany). PCR products were purified, digested with *FseI *and *AscI* (NEB Inc, MA, USA), and then cloned into the pCS2^+^ vector. For the synthesis of cRNA, plasmids were linearized by NotI. pAd‐Track Flag‐SIRT1 was purchased from addgene. Capped cRNAs were made using in vitro transcription with SP6 mMESSAGE mMACHINE (Ambion, CA, USA) according to the manufacturer's instruction. Synthesized RNA was aliquoted and stored at −80°C. The related primer sequences can be found in Supporting Information Table S1.

### 4.5 NMNAT2 knockdown and overexpression analysis

Microinjections of siRNA or mRNA were used to knockdown or overexpress proteins in mouse oocytes, respectively. Nmnat2‐targeting siRNA was diluted with RNase‐free water to give a stock concentration of 1 mM, and 2.5 pl solution was injected. 10 pl cRNA solution (10 ng/µl) was injected into oocytes in overexpression experiments. Following injections, oocytes were arrested at GV stage in medium containing 2.5 μM milrinone for 20 hr to promote mRNA degradation or translation. The related primer sequences can be found in Supporting Information Table S1.

### 4.6 Immunoblotting

A total of 200 oocytes were lysed in Laemmli buffer containing protease inhibitor. Proteins were separated by SDS‐PAGE and then transferred to PVDF membranes. Membranes were blocked in TBS containing 0.1% Tween 20 and 5% low fat dry milk for 1 hr and then incubated overnight at 4°C with primary antibodies: mouse anti‐SIRT1 antibody (1:1,000) or mouse anti‐NMNAT2 antibody (1:1,500). After multiple washes in TBS containing 0.1% Tween 20 and incubation with HRP‐conjugated secondary antibodies, the protein bands were visualized using an ECL Plus Western Blotting Detection System. The membrane was then washed and reblotted with anti‐β‐actin antibody (1:10,000) for loading control.

### 4.7 Immunofluorescence

Oocytes were fixed with 4% paraformaldehyde for 30 min and then permeabilized with 0.5% Triton X‐100 for 20 min. Following treatment with blocking solution (1% BSA‐supplemented PBS) for 1 hr, samples were incubated overnight at 4°C with primary antibodies. To visualize spindle, oocytes were probed with FITC‐conjugated tubulin antibody. Chromosomes were evaluated by staining with propidium iodide (PI) or Hoechst 33342 for 10 min. After washed in PBS, oocyte were mounted on anti‐fade medium (Vectashield, Burlingame, CA, USA) and examined under a laser scanning confocal microscope (LSM 710, Zeiss, Germany).

Fluorescence intensity was assessed as we described previously (Han et al., 2018). When quantifying ROS signal, a Z‐stack of oocyte was taken, and images were used to generate a projection containing all ROS signals. Acquisition settings were not altered throughout the experiment. ROS signal was finally calculated as the mean fluorescence intensity (measured from total cytoplasmic intensity and normalized to cell area using ImageJ), following background subtraction from an equal region in the negative control oocyte. To quantify H4K16ac staining in oocytes, fluorescence was measured and normalized to DNA intensity.

### 4.8 Measurement of NAD^+^ levels

Measurement of NAD^+^ levels is conducted using a commercially available kit (Sigma, St. Louis, MO; MAK037) according to the manufacturer protocol. In brief, 150 oocytes were harvested for total NAD^+^ extraction and quantification based on the procedure described by Pantazi et al. (2015). The NAD^+^ concentration is calculated by subtracting the NADH values from NAD_total _(NAD^+^ and NADH). NAD_total_ and NADH levels are quantified in a colorimetric assay at 450 nm using iMark™ Microplate Absorbance Reader (BIO‐RAD).

### 4.9 ROS evaluation

To detect intercellular ROS in living oocytes, CM‐H2DCFDA from Invitrogen was used. CM‐H2DCFDA was prepared in DMSO prior to loading. Oocytes were incubated with 5 μM CM‐H2DCFDA for 30 min at 37°C, and then immediately observed under laser scanning confocal microscope (LSM 710, Zeiss, Germany).

### 4.10 Determination of ATP levels

Total ATP content was determined using the bioluminescent somatic cell assay kit (Sigma, MO, USA). Briefly, 20 oocytes were pooled together and processed according to the procedure we previously published (Hou et al., 2015). A 6‐point standard curve (0, 0. 1, 0.5, 1.0, 10, and 50 pmol of ATP) was generated in each assay, and the ATP content was calculated by using the formula derived from the linear regression of the standard curve.

### 4.11 Chromosome spread

Chromosome preparations for MII oocytes were performed as described previously (Li et al., 2017). MII oocytes were exposed to Tyrode's buffer (pH 2.5) for 30 s at 37°C to remove zona pellucida. Oocytes were fixed in a drop of 1% paraformaldehyde with 0.15% Triton X‐100 on a glass slide. After air drying, oocytes were incubated with CREST (1:500) overnight at 4°C and then Cy5‐conjugated secondary antibody for 1 hr for kinetochore labeling. Samples were examined under a laser scanning confocal microscope.

### 4.12 Statistical analysis

Data are presented as mean ± *SD*, unless otherwise indicated. Differences between two groups were analyzed by Student's *t* test. Multiple comparisons between more than two groups were analyzed by one‐way ANOVA test using Prism 5.0. *p* < 0.05 was considered to be significant.

## CONFLICT OF INTEREST

None declared.

## AUTHORS' CONTRIBUTION

XW and QW designed research; XW, JZ, DQ, LH, HW, and JG performed research; XW, FH, XY, and QW analyzed data; XW and QW wrote paper.
