# Supplementary material for: NMNAT2‐mediated NAD+ generation is essential for quality control of aged oocytes
Source: Aging Cell. 2019 Mar 25;18(3):e12955. doi: 10.1111/acel.12955 (PMC6516161; doi:10.1111/acel.12955)
Supplement: Supplementary file 1 [file ACEL-18-e12955-s001.docx]

# Supplemental Table 1

# Primer sequences of gene for cloning

| ***Gene*** | ***Primer sequence*** |
| --- | --- |
| Nmnat2 | F:5′- GGGGGCCGGCCGATGACCGAGACCACAAAG -3′  R:5′- GGGGGCGCGCCAGGACACGGGGAGAATAGT -3′ |

# Primer sequences of genes for qRT-PCR

| ***Gene*** | ***Primer sequence*** |
| --- | --- |
| GAPDH  Nampt  Nrk1  Nrk2  Nmnat1  Nmnat2  Nmnat3 | F: 5’ –CTTTGTCAAGCTCATTTCCTGG – 3’  R: 5’ –TCTTGCTCAGTGTCCTTGC – 3’  F: 5’ –AACCAATGGCCTTGGGGTTA– 3’  R: 5’ –AACCAATGGCCTTGGGGTTA– 3’  F: 5’ –CTTGAAGCTTGCTCTGCGAC– 3’  R: 5’ –TAGCGCTTCAAGCACATCAT– 3’  F: 5’ –ACGGGGTGGAAGTGGTCTAT– 3’  R: 5’ –TAGGGACCATACAGGACGCC– 3’  F: 5’ –CCTTCAAGGCCTGACAACAT– 3’  R: 5’ –ACCGACCGGTGAGATAATGC– 3’  F: 5’ –GATGTTCGAGAGAGCCAGGG– 3’  R: 5’ –GTCGGAATTCTGGACAGCCA– 3’  F: 5’ –TGTCCACGAAGCCTTGAGTC– 3’  R: 5’ –GCAGTGGCCACCCTGTTTTA– 3’ |

# Nmnat2 siRNA and control siRNA sequences

| Nmnat2  F: 5’-GCAUGCCCUUUACCACAAATT-3’  R :5’-UUUGUGGUAAAGGGCAUGCTT-3’  Negative control sequence :  F: 5’-UUC UCC GAA CGU GUC ACG UTT-3’  R:5’-ACG UGA CAC GUU CGG AGA ATT-3’ |
| --- |
